# Carnitine derivatives beyond fatigue: an update

**DOI:** 10.1097/MOG.0000000000000906

**Published:** 2023-01-05

**Authors:** Michele Malaguarnera, Vito Emanuele Catania, Mariano Malaguarnera

**Affiliations:** a Universidad Europea de Valencia, Faculty of Health Science, Valencia, Spain; b Department of Medical, Surgical Sciences and Advanced Technologies ‘G.F. Ingrassia’; c Research Centre ‘The Great Senescence’, University of Catania, Catania, Italy

**Keywords:** carnitine, chronic liver disease, fatigue, frailty, hepatocellular carcinoma, metabolic flexibility, mitochondrial dysfunction, nonalcoholic fatty liver disease

## Abstract

**Purpose of review:**

Carnitine is an essential micronutrient that transfer long-chain fatty acids from the cytoplasm into the mitochondrial matrix for the β-oxidation. Carnitine is also needed for the mitochondrial efflux of acyl groups in the cases wherein substrate oxidation exceeds energy demands.

**Recent findings:**

Carnitine deficiency can affect the oxidation of free fatty acids in the mitochondria resulting in the aggregation of lipids in the cytoplasm instead of entering the citric acid cycle. The aggregation leads a lack of energy, acetyl coenzyme A accumulation in the mitochondria and cytotoxic production.

**Summary:**

Carnitine and its derivatives show great clinical therapeutic effect without significant side effects.

## INTRODUCTION

Carnitine is a trimethylated amino acid composed by lysine and methionine, ubiquitously present in every human cell and has an important role in energy metabolism and production of cellular energy. Carnitine has been extensively used in various research activities to obtain beneficial effects under pathological states. Metabolically, carnitine plays an important role in energy generation by fatty acid oxidation in the mitochondrial matrix. Moreover, carnitine has an acyl group acceptor that facilitate mitochondrial exports of the carbon in excess forming of acyl-carnitine.

Its physiological role is associated with the cellular energy production through the transport of long chain fatty acids from the cytosol into the mitochondria where their degradation takes place via β-oxidation. This function is allowed by the constitution of a complex with activated fatty acid (acyl-Coa), termed as ‘Carnitine shuttle’, that facilitates the elimination of oxidative products [1] and other toxic fatty metabolites

Carnitine also show the ability to inhibit the apoptosis and to improve the function of the bone marrow progenitors, by increasing the number of colony-forming units improving thrombocytopoiesis, erythropoiesis and leucopoiesis [2]. 

**Box 1:**
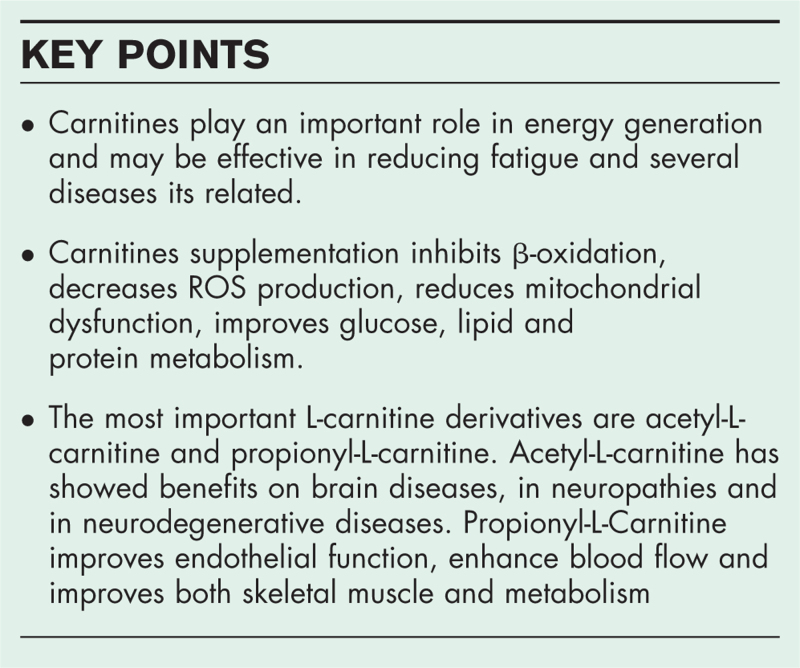
no caption available

## CARNITINE, MITOCHONDRIA AND METABOLIC FLEXIBILITY

The charged tri-methylamine group and the carboxylic group of L-carnitine permit the interaction with the membrane phospholipids, glycolipids and proteins. As a consequence, carnitine contribute to the maintain of the stability of different compartments such as mitochondria, cellular membrane and other organelles [3^▪^]. The mitochondria membrane stability is crucial for many processes, including the cellular survival. Indeed, mitochondria performs various functions, including cellular respiration and energy production. Moreover, cellular responses to the changes in nutritional state are assigned to the mitochondria.

Mitochondrial dysfunction arises from an inadequate number of mitochondrial, an inability to provide necessary substrates to mitochondria or a loss of efficiency in the electron transport chain and consequent reduction of ATP synthesis [4].

Mitochondrial dysfunction may underly cellular senescence, when cells accumulate damage and lose their metabolic flexibility. When cellular metabolism is efficient, senescent cells are removed by apoptosis. If this process does not take place, it can result in morbid status like kidney, liver, heart or neurodegenerative diseases.

Carnitine is considered a key molecule associated with the mechanism of metabolic flexibility, as it regulates Acyl-COA and acetyl COA and preserves free CoA levels within the mitochondria.

Metabolic flexibility represents the capacity to switch from oxidation while fasting to carbohydrate oxidation in the insulin-stimulated state [5]. It is linked to the capacity of mitochondria to select fuel in response to nutritional changes and placed mitochondrial function at its core [6,7].

## CARNITINE IN FATIGUE

Fatigue can be a constant or a temporary state reducing energy levels, concentration and motivation. It is often a sign of a serious disease such as cancer, autoimmune diseases and infections. This condition has been associated with irregularities in the metabolism [8].

The carnitine plays a major role in the metabolism of fatty acids and its inadequacy will induce feelings of tiredness or general fatigue [9,10].

In addition, more than 95% of the body's total carnitine is located in muscle and it is essential for the transportation of long-chain fatty acids through the mitochondrial membrane for beta oxidation and energy production in the form of ATP [11].

The link between mitochondrial ATP formation and cytosolic processes involves adenine nucleotide transporter, mitochondrial kinases at the contact sites between the contact sites between the outer and inner mitochondrial membranes.

Fatigue may be induced by various causes, including physical and mental stress, inflammation, viral infection and neurological diseases. The mechanism involved with fatigue include increase energy requirement, reduced availability of substrates, atypical generation of compounds that disrupt metabolic homeostasis and regular function of various organs. In this context, the impaired synthesis, transport or metabolism of carnitine can lead to early or secondary deficiencies.

## FRAILTY AND CARNITINE

Frailty is a condition characterized by an increase of vulnerability to exogenous or endogenous stress and represents the gradual decline of functions, loss of autonomy and a wide spectrum of adverse outcomes. It is characterized by muscle weakness, sarcopenia and fatigue [12].

These adverse outcomes may include increased hazard for death, hospitalization, falls, worsening activities of daily living (ADL), disability, dependency and need for long-term care [13,14]. However, this progression could be reversible in some patients.

Frailty predicts future disability, but might be modifiable, particularly at an early stage.

Recently, carnitine has been found diminished in frailty elderly patients and that the supplementation with the acetylated derivative, Acetyl-L-carnitine (ALCAR), improve physical and cognitive processes [15,16]. The possibility of providing acetyl groups makes ALCAR able to maintain the intramitochondrial pathways, to reactivate coenzyme A, to reduce peroxidation and intracellular malonyl aldehyde levels, to act as a scavenger and to contribute to neurotransmitter synthesis due to the structural affinity to acetylcholine [17].

## CARNITINE AND LIVER

Liver is the main site for L-carnitine. Reduced levels of carnitine in patients with liver disease may negatively impact on fatty acid oxidation, also increasing ROS production and contributing to mitochondrial impairment. This organ plays a central role in the regulation of energy metabolism and in the adoption to both nutrients and micronutrients and macronutrients availability.

Maintaining energy homeostasis requires substrate sensing, trafficking storage and utilization, dependent on substrate availability and energy requirement [4,18].

L-carnitine and its derivates have been used as adjuvants therapies in liver disease, including nonalcoholic steatohepatitis in alcoholic fatty liver disease, viral hepatitis and cirrhosis [19–23].

The treatment with carnitine associated with entecavir reduced the levels of alanine transaminase in chronic hepatitis B patients [24]. A recent observational study also suggests an immunomodulation activity by L-carnitine that could be helpful in the treatment of HBV [25].

Also, a recent study has shown that changes in serum-free carnitine levels after hepatitis C virus eradication by directly acting antivirals are associated with increased muscle mass [26].

In general, the carnitine supplementation may contribute to reduce muscle loss and reduces inflammation and oxidative stress.

Supplementation of carnitine and its derivates have also showed a favourable effect in hepatic encephalopathy reducing serum ammonia and increase albumin and nutritional status and improve both cognitive and motor functions [27].

Because L-carnitine has the potential to counteract mitochondria dysfunction, carnitine supplementation not only contribute to inhibits effects on sarcopenia but also may obtain clinical efficacy to the treatment failure, progression-free survival, post progression survival and overall survival in hepatocellular carcinoma [28,29^▪^,^30^^▪▪^,^31^,^32^].

## CONCLUSION

L-carnitine and its derivatives play a critical role in metabolic functions, including fatty acid metabolism and the production of ATP. Carnitine supplementation improves the functions of mitochondria, reduces fatigue and inflammations processes.

The carnitine supplementation and its derivatives have shown to counteract mitochondria dysfunction and the metabolic inflexibility. Carnitine not only reduces fatigue and frailty but also represents a protective role in acute, chronic liver diseases, in ALD and in NAFLD. Furthermore, L-carnitine treatment decrease the progression and the development in cirrhosis complications and in hepatocellular carcinoma.

## Acknowledgements

*None.*

### Financial support and sponsorship

*None.*

### Conflicts of interest

*There are no conflicts of interest.*

## REFERENCES AND RECOMMENDED READING

Papers of particular interest, published within the annual period of review, have been highlighted as:

▪ of special interest
▪▪ of outstanding interest
